# Protective Effects of *Lactobacillus plantarum* CCFM8610 against Acute Toxicity Caused by Different Food-Derived Forms of Cadmium in Mice

**DOI:** 10.3390/ijms222011045

**Published:** 2021-10-13

**Authors:** Jiamin Zhu, Leilei Yu, Xudan Shen, Fengwei Tian, Jianxin Zhao, Hao Zhang, Wei Chen, Qixiao Zhai

**Affiliations:** 1 State Key Laboratory of Food Science and Technology, Jiangnan University, Wuxi 214122, China; mike64@126.com (J.Z.); edyulei@126.com (L.Y.); ewingshen@163.com (X.S.); fwtian@jiangnan.edu.cn (F.T.); zhaojianxin@jiangnan.edu.cn (J.Z.); zhanghao61@jiangnan.edu.cn (H.Z.); chenwei66@jiangnan.edu.cn (W.C.); 2 School of Food Science and Technology, Jiangnan University, Wuxi 214122, China; 3 National Engineering Research Center for Functional Food, Jiangnan University, Wuxi 214122, China; 4 Wuxi Translational Medicine Research Center and Jiangsu Translational Medicine Research Institute, Wuxi Branch, Wuxi 214122, China

**Keywords:** food–derived cadmium, probiotics, acute toxicity, gut microbiota, serum metabolomics

## Abstract

Cadmium (Cd) is an environmental pollutant that is toxic to almost every human organ. Oral supplementation with lactic acid bacteria (LAB) has been reported to alleviate cadmium toxicity. However, research on the mitigation of cadmium toxicity by LAB is still limited to inorganic cadmium, which is not representative of the varied forms of cadmium ingested daily. In this study, different foodborne forms of cadmium were adopted to establish an in vivo toxicity model, including cadmium–glutathione, cadmium–citrate, and cadmium–metallothionein. The ability of *Lactobacillus plantarum* CCFM8610 to reduce the toxic effects of these forms of cadmium was further investigated. The 16S rRNA gene sequencing and metabolomics technologies based on liquid chromatography with tandem mass spectrometry (LC–MS/MS) were adopted for the exploration of relevant protective mechanisms. The results demonstrated that the consumption of CCFM8610 can reduce the content of cadmium in mice and relieve the oxidative stress caused by different food–derived forms of cadmium, indicating that CCFM8610 has a promising effect on the remediation of the toxic effects of cadmium food poisoning. Meanwhile, protective effects on gut microflora and serum metabolites might be an important mechanism for probiotics to alleviate cadmium toxicity. This study provides a theoretical basis for the application of *L. plantarum* CCFM8610 to alleviate human cadmium poisoning.

## 1. Introduction

Cadmium is a highly toxic heavy metal that is commonly found in industrial pollutants. There are a large variety of sources of Cd exposure in humans, which include the intake of cadmium–contaminated food, water, and tobacco products [1]. Because of its high ability to bind metallothionein (MT) in the body, Cd has various toxic effects on the kidney, liver, bone, lung, cardiovascular system, testis, and endocrine system [2]. Cd and its compounds have been listed as the seventh most hazardous substance to health by the Agency for Toxic Substances and Disease Registry [3], and are also listed as first–class carcinogens by the International Agency for Research on Cancer [4].

Several studies have advocated for moderate nutritional intervention or dietary therapy to prevent and control Cd poisoning, mainly including supplementation of trace elements, vitamins, and extracts of fruits and vegetables. The main mechanisms are the reduction in Cd accumulation in vivo and the remission of oxidative stress in tissues. Sokkary et al. explored the protective effects of vitamin C on Cd toxicity in the lungs and brains of rats and found that the intake of vitamin C could relieve oxidative damage and morphological changes [5]. Nemmiche also proposed that cadmium could induce the oxidation of cellular lipids and proteins, and the application of α–tocopherol could reduce the oxidative stress induced by cadmium and increase the level of glutathione and other biochemical parameters [6].

Studies have shown that some lactic acid bacteria (LAB) can bind and remove Cd in vitro. Halttunen et al. assessed the ability of specific LAB to adsorb Cd and lead in water and observed remarkable removal efficiency [7]. Probiotic LAB has also been found to have antioxidative functions, which can effectively relieve the oxidative stress caused by Cd exposure in vivo. Jafarpour et al. used a symbiotic diet containing probiotics (*Lactobacillus plantarum* and *Bacillus coagulans*) and prebiotics (inulin) to treat acute Cd–exposed rats and found that it could protect the liver and kidneys by reducing Cd accumulation, preventing tissue damage, and restoring antioxidant enzyme activity [8]. Considering these special functions, LAB consumption might be a promising protective approach for populations exposed to Cd.

Although probiotics have a strong potential to alleviate Cd toxicity, research on their mitigation of cadmium toxicity is still limited to some inorganic Cd, such as CdCl_2_ and Cd(NO_3_)_2_, which do not represent the forms of Cd ingested daily. Studies have shown that Cd can combine with proteins, organic acids, and be in an ionic state [9,10]. Zhao et al. used HPLC with ICP–MS to explore the main forms of Cd in marine shellfish and found that Cd mainly existed in the forms of Cd–MT, Cd–Cysteine (Cd–Cys), and Cd–glutathione (Cd–GSH) [11]. Yang et al. explored the speciation of Cd in Indian mustard using HPLC–ICP–MS. The results showed that Cd exists in the forms of Cd–(phytochelatin)_3_, Cd–(phytochelatin)_2_, Cd–GSH, and Cd–Cys in Indian mustard. Previous studies have shown that different forms of Cd could result in unequal acute toxin influence [12]. Koropatnick [13] treated mice with Cd–MT and CdSO_4_ separately and found that Cd–MT mainly improved the renal Cd content, while a specific increase in the hepatic Cd content was observed in the CdSO_4_–treated group. Our previous study also showed that diverse forms of Cd can result in diverse acute toxicity effects in mice [14]. Therefore, it is essential to investigate the bioreduction function of probiotics on different foodborne forms of Cd, which can evaluate the protective effect of probiotics on Cd toxicity in humans with more precision.

In our previous studies, we found a probiotic strain, *Lactobacillus plantarum* CCFM8610, which could efficiently bind CdCl_2_ and relieve the acute and chronic toxin damage induced by CdCl_2_ in mice [15,16]. In this study, we investigated the capability of CCFM8610 to reduce the toxic effects of different foodborne forms of Cd. Cd–citrate, Cd–GSH, and Cd–MT were used to represent the forms of Cd in Cd–containing foods. Meanwhile, 16S rRNA gene sequencing technology and metabolomics technologies based on LC–MS were adopted for further exploration of the relevant protective mechanisms.

## 2. Results

### 2.1. Changes in Cadmium Content and Antioxidant Indexes in Livers and Kidneys

**Figure 1:**
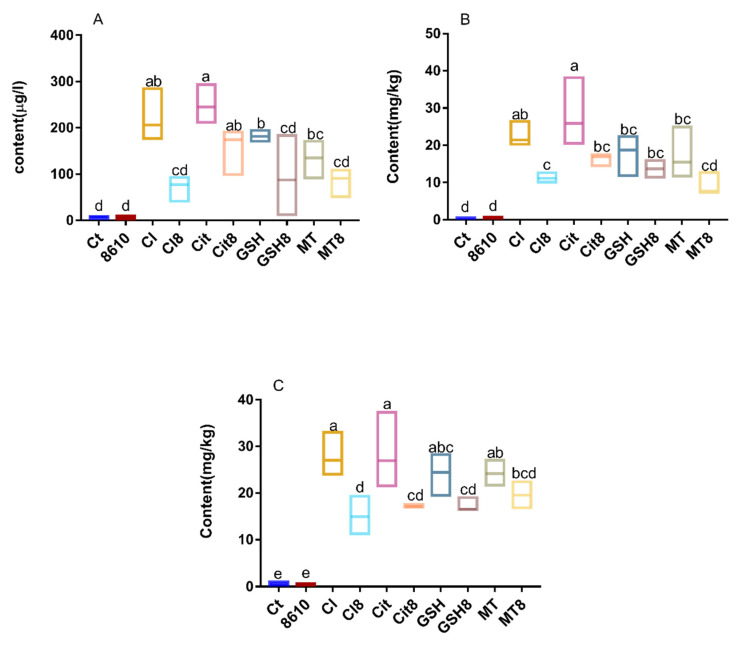
Changes in cadmium content in the blood (**A**), liver (**B**), and kidneys (**C**) of mice. Ct, Control; 8610, CCFM8610 treatment only; Cl, CdCl_2_; Cl8, CdCl_2_ + CCFM8610; Cit, Cd–citrate; Cit8, Cd–citrate + CCFM8610; GSH, Cd–glutathione (Cd–GSH); GSH8, Cd–GSH + CCFM8610; MT, Cd–Metallothionein (Cd–MT); MT8, Cd–MT + 8610. Different small letters in the figures indicate significant differences at *p* < 0.05. See also in following figures.

**Figure 2:**
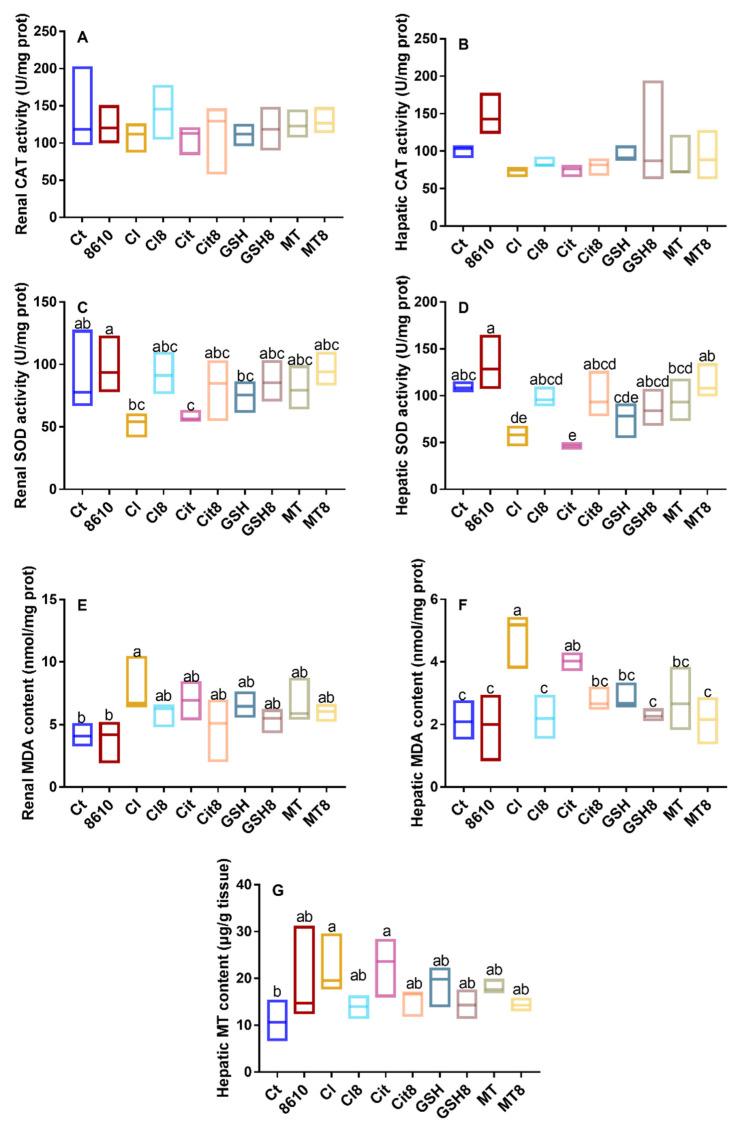
Changes in antioxidant indexes in the liver and kidneys of mice. (**A**). CAT in the kidneys. (**B**). CAT in the liver. (**C**). SOD in the kidneys. (**D**). SOD in the liver. (**E**). MDA in the kidneys. (**F**). MDA in the liver. (**G**). MT in the liver.

Diverse accumulation of Cd in tissues and blood were observed in different groups (Figure 1), indicating that there are differences in the bioabsorption and bioaccumulation of these forms of Cd. The antioxidant indexes in the tissues are shown in Figure 2. Different foodborne forms of Cd could reduce the activity of superoxide dismutase (SOD) in the liver and kidneys, while less damage was observed in mice treated with Cd–MT and Cd–GSH. Similarities can be seen in the concentrations of malondialdehyde (MDA) and MT, as well as CAT activity. In summary, mice treated with CdCl_2_ and Cd–citrate suffered more damage to the body than the other treatment groups, which is consistent with our previous studies [14].

The consumption of CCFM8610 can reduce the content of Cd and relieve the oxidative stress resulting from the acute administration of different foodborne forms of Cd. As illustrated in Figure 1 and Figure 2, CCFM8610 markedly inhibited the accumulation of Cd in the liver and kidneys of the CdCl_2_ and Cd–citrate treatment groups. A decreasing trend of Cd concentration in the tissues and blood was also observed in the Cd–GSH and Cd–MT treatment groups. Similarities can be found in the recovery of the antioxidant system, indicating that the administration of CCFM8610 has a protective effect on mice treated with different foodborne forms of Cd.

### 2.2. Changes in the Composition of the Intestinal Microbiota

**Figure 3:**
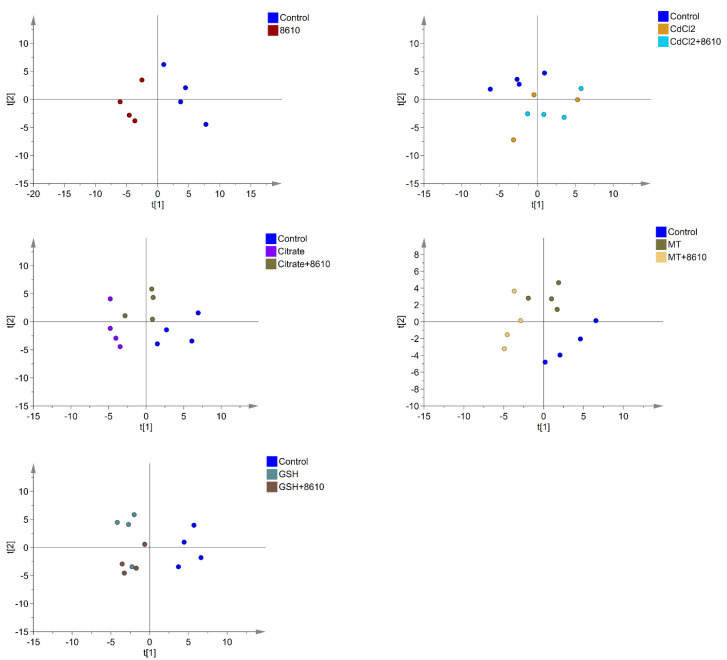
Microbiome multivariate analysis based on Principal Component Analysis (PCA) of fecal microbiota in mice.

**Figure 4:**
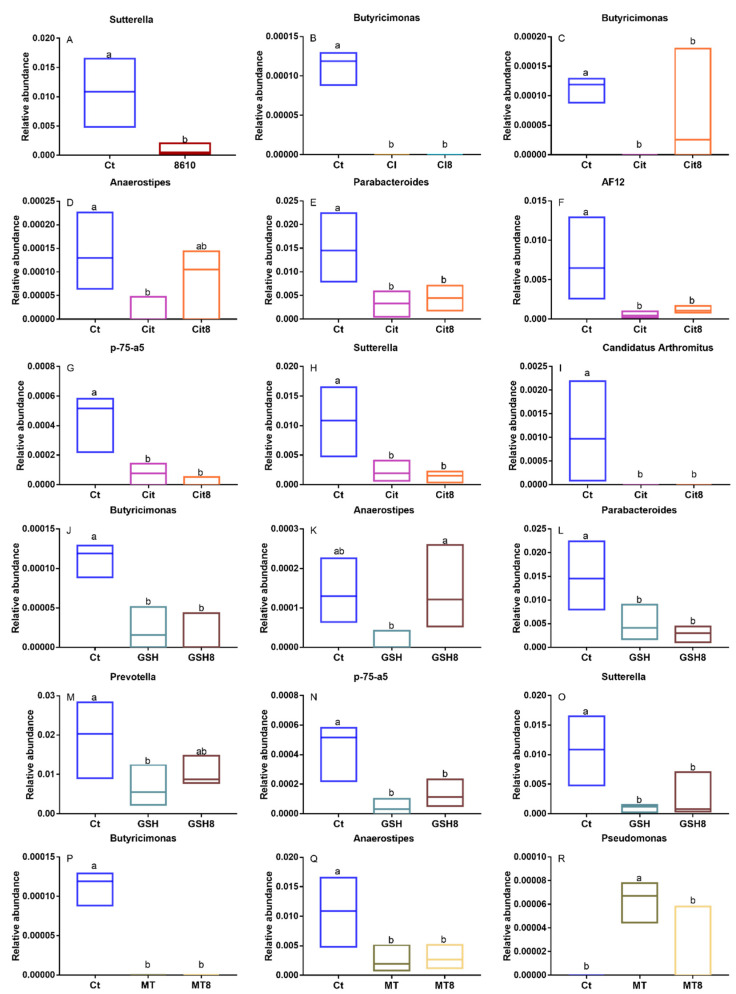
Analysis of the changes in the abundance of microflora in different groups. (**A**,**B**). Changes in CdCl_2_ treatment groups. (**C**–**I**). Changes in Cd–citrate treatment groups. (**J**–**O**). Changes in Cd–GSH treatment groups. (**P**–**R**). Changes in Cd–MT treatment groups.

The microbiome of 39 fecal samples (one sample in the CdCl_2_ groups was absent due to the failure in fecal collection) were analyzed by 16S rRNA gene sequencing technology. The PCA score plot has shown that there were obvious differences between control and Cd–treatment groups in the intestinal microbiota of mice (Figure 3). LDA effect size (LEfSe) analysis and significance analysis were performed for the biomarkers among the groups (Figure 4 and Figure S1–S5). By comparing the control and the Cd–treated mice, the different foodborne forms of Cd all resulted in a decrease in *Butyricimonas*, which shows that the genus was sensitive to Cd exposure. Additionally, different foodborne forms of Cd can cause diverse changes in the intestinal microbiota. For example, *Anaerostipes* decreased significantly in all Cd–treated groups, except in the CdCl_2_ treatment group. Acute exposure to Cd–citrate resulted in a decrease in *Parabacteroides*, p75–a5, and *Sutterella*. Moreover, Cd–GSH treatment decreased the ratio of *Prevotella*.

The intake of CCFM8610 altered the diversity of the intestinal flora in mice. The ratio of the genera affected to the Cd exposure was determined after the CCFM8610 treatment. The abundance of *Butyricimonas* and *Anaerostipes* in the Cd–citrate + CCFM8610 group increased markedly, and a similar trend was observed in the abundance of *Anaerostipes* and *Prevotella* in the Cd–GSH + CCFM8610 group. Moreover, the abundance of *Pseudomonas* in the Cd–MT + CCFM8610 group also increased significantly. Considering CCFM8610′s function of reducing cadmium accumulation and alleviating oxidative stress in vivo, changes in the gut microbiota indicate that CCFM8610 can alleviate Cd toxicity by regulating the structure of the intestinal flora.

### 2.3. Changes in Serum Metabolites

**Figure 5:**
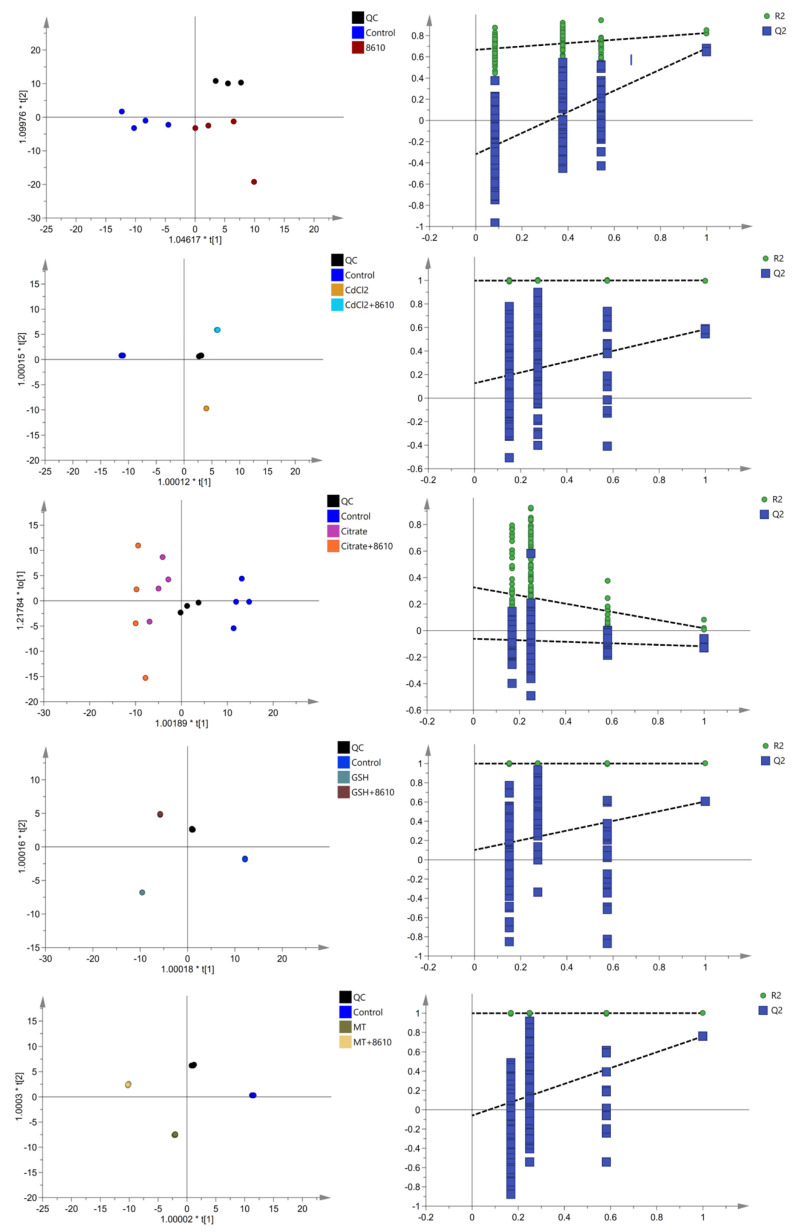
A plot of OPLS–DA scores of the different groups based on their metabolites.

The blood metabolites of 38 serum samples (one sample in CdCl_2_ groups and one sample in Cd–GSH group were absent due to the hemolysis during serum separation) were analyzed. The change in serum metabolites was similar to that in the intestinal flora. Difference in the composition of serum metabolites of mice between control and Cd–treatment groups can be observed through the OPLS––DA score plots (Figure 5). By comparing the change in the abundance of serum metabolites in different Cd–treatment groups with the control group, we found that different forms of cadmium resulted in diverse changes (Table S1). For example, CdCl_2_ increased the abundance of sphingosine and beta––d––Fructose 6––phosphate and decreased the abundance of 8,11,14––Eicosatrienoic acid, adrenic acid, docosahexaenoic acid, eicosapentaenoic acid, elaidic acid, ethyl dodecanoate, and ethyl tetradecanoate, while Cd–MT decreased the abundance of spermidine, citrulline, ethyl tetradecanoate, pelargonic acid, and 2,6––Di––tert––butyl––4––hydroxymethylphenol.

The administration of CCFM8610 could regulate the abundance of some serum metabolites, which changed significantly after the exposure to different foodborne forms of cadmium. In detail, CCFM8610 treatment increased the abundance of docosahexaenoic acid, elaidic acid, ethyl dodecanoate, ethyl tetradecanoate, and 11,12––EpETrE, which were shown to decrease after exposure to CdCl_2_. The abundance of sphingosine and indoxyl sulfate were decreased in Cd–citrate + CCFM8610 group, while the abundance of 3––methylhistamine, l–acetylcarnitine, uric acid, and d–galactose were increased. Similar changes could be observed in the abundance of Histamine, Methionine sulfoxide, 2–Furoic acid, Docosahexaenoic acid, Eicosapentaenoic acid, Ethyl dodecanoate, Oxoglutaric acid, 2,6–Di–tert–butyl–4–hydroxymethylphenol, and d–Galactose the in Cd–GSH + 8610 group and the abundance of Spermidine, Pantothenic acid, 2,6–Di–tert–butyl–4–hydroxymethylphenol in the Cd–MT + 8610 group.

### 2.4. Correlation Analysis between Intestinal Microbiota and Serum Metabolites

**Figure 6:**
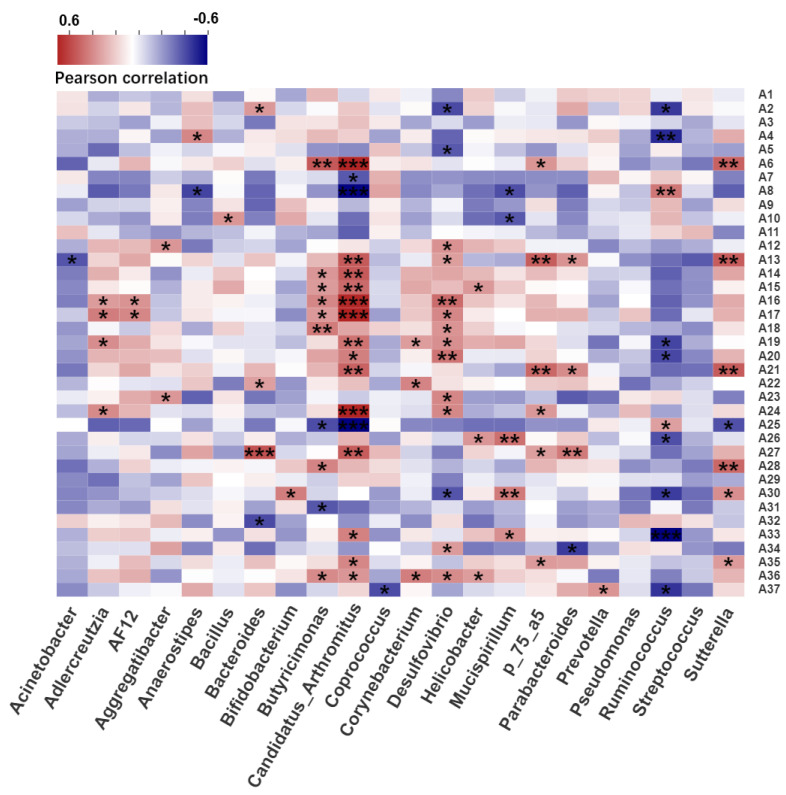
Pearson correlation analysis between the intestinal flora and serum metabolites. A1, 3–Methylhistamine; A2, Acetylcholine; A3, Histamine; A4, Spermidine; A5, Sphingosine; A6, Citrulline; A7, l–Arginine; A8, l–Glutamic acid; A9, l–Methionine; A10, Methionine sulfoxide; A11, Taurine; A12, 16–Hydroxy hexadecanoic acid; A13, 2–Furoic acid; A14, 8,11,14–Eicosatrienoic acid; A15, Adrenic acid; A16, Docosahexaenoic acid; A17, Eicosapentaenoic acid; A18, Elaidic acid; A19, Ethyl dodecanoate; A20, Ethyl tetradecanoate; A21, Oxoglutaric acid; A22, Pelargonic acid; A23, Ginkgoic acid; A24, Urocanic acid; A25, Beta–d–Fructose 6–phosphate; A26, Ribose 1–phosphate; A27, l–Acetylcarnitine; A28, Pantothenic acid; A29, l–Norleucine; A30, Beta–d–Glucopyranuronic acid; A31, Indoxyl sulfate; A32, Corticosterone; A33, 2,6–Di–tert–butyl–4–hydroxymethylphenol; A34, 11,12–EpETrE; A35, Uric acid; A36, d–Galactose; A37, Sulfate. * *p* < 0.05; ** *p* < 0.01; *** *p* < 0.001.

**Figure 7:**
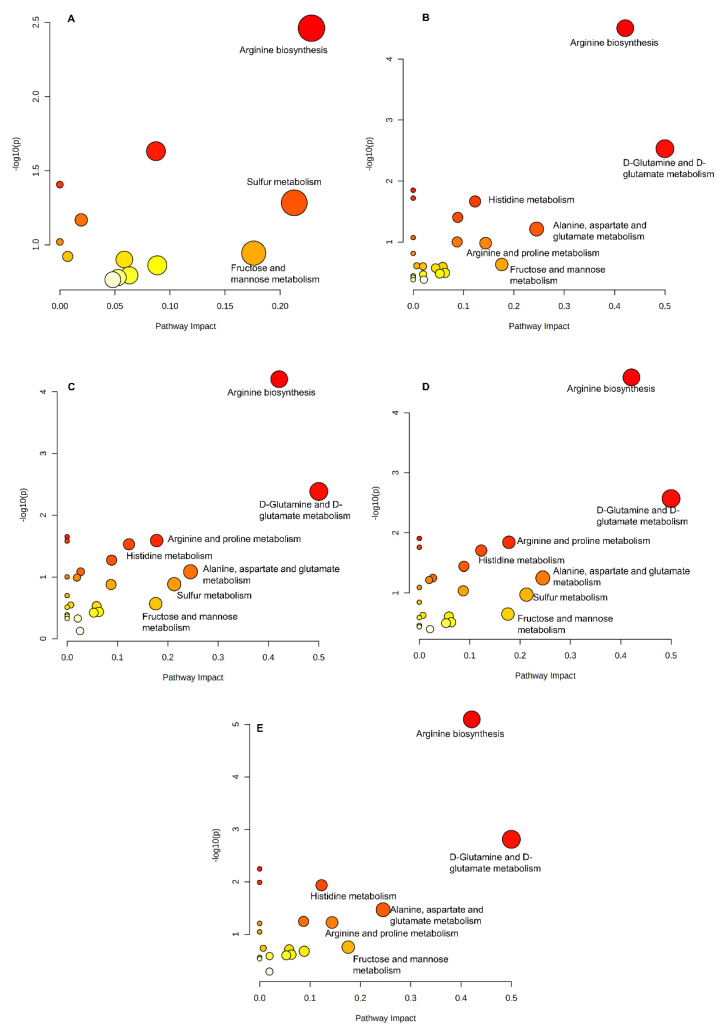
Summary of pathways analysis of mice with CCFM8610 treatment with MetaboAnalyst. (**A**), CCFM8610 only treatment groups; (**B**), CdCl_2_ treatment groups; (**C**), Cd–citrate treatment groups; (**D**), Cd–GSH treatment groups; (**E**), Cd–MT treatment groups.

Pearson correlation analysis was used to determine the relationship between intestinal flora and serum metabolites (Figure 6). The genera *Butyricimonas, AF12*, *Candidatus Arthromitus*, *Desulfovibrio*, and *Helicobacter*, which were considered as the biomarkers for CdCl_2_ exposure, were calculated using LEfSe analysis in the CdCl_2_ treatment groups. These genera had a significant correlation with uric acid, pyroglutamic acid, pelargonic acid, pantothenic acid, oxoglutaric acid, methionine sulfoxide, l–phenylalanine, l–norleucine, l–glutamic acid, l–acetylcarnitine, ginkgoic acid, ethyl tetradecanoate, ethyl dodecanoate, elaidic acid, cis–5,8,11,14,17–eicosapentaenoic acid, docosahexaenoic acid, d–galactose, citrulline, beta–d–fructose 6–phosphate, arachidonic acid, adrenic acid, 8,11,14–eicosatrienoic acid, 3–methylhistamine, 2–furoic acid, 2,6–di–tert–butyl–4–hydroxymethylphenol, 1–phenyl–1,3–octadecanedione, 16–hydroxy hexadecanoic acid, and 11,12–EpETrE. The KEGG pathway results indicated that the metabolites were mainly involved in arginine biosynthesis, d–glutamine and d–glutamate metabolism, phenylalanine, tyrosine, and tryptophan biosynthesis, arginine, alanine, aspartate, and glutamate metabolism, arachidonic acid metabolism, fructose and mannose metabolism, and phenylalanine metabolism (Figure 7). In summary, arginine biosynthesis, d–glutamine and d–glutamate metabolism, and fructose and mannose metabolism participated in all groups, which indicated that these pathways are sensitive to cadmium exposure. Additionally, different pathways could also be found among the groups, such as sulfur metabolism in the Cd–GSH treatment groups and phenylalanine metabolism in the CdCl_2_ treatment groups.

## 3. Discussion

Former studies showed that probiotics could relieve cadmium toxicity mainly by reducing the absorption of cadmium in the body [15,17,18,19] and relieving the oxidative stress caused by cadmium exposure [20]. As Cd in food does not exist in inorganic form, foodborne forms of Cd should be considered when exploring measures to alleviate toxicity effects caused by the intake of Cd–containing food. In this study, different foodborne forms of Cd were used to investigate the ability of CCFM8610 to mitigate their toxic effects. By comparing the Cd–treatment groups and CCFM8610–treatment groups, the administration of CCFM8610 alleviated the toxic effects caused by different foodborne forms of Cd, which proves that CCFM8610 has broad application potential in alleviating Cd food poisoning.

Heavy metals are mainly excreted from the body through feces, which indicates that the heavy metals in the gastrointestinal tract could change the composition of the gut flora [21]. Lu et al. treated mice with As at a concentration of 10 ppm for four weeks and evidenced significant changes in the gut microbiome [22]. Liu et al. also found that Cd treatment could reduce the intestinal flora diversity and change the Firmicutes/Bacteroidetes ratio [23]. Zhang et al. treated mice with CdCl_2_ at a concentration of 10 mg/L and observed marked decreases in Firmicutes [24]. These results indicate that exposure to Cd perturbs intestinal bacteria [25]. The present study is the first attempt to analyze the changes in intestinal microbiota in mice treated with different foodborne forms of Cd based on 16S rRNA gene sequencing. It is worth noting that different foodborne forms of Cd resulted in distinct changes in the gut microbiome composition of the mice. These changes might involve different metabolic pathways of intestinal microorganisms, indicating that the effect of Cd on the microbiome should be discussed separately according to its different binding forms.

In previous studies, probiotics have been shown to protect against changes in the bacterial population structure caused by external pressure. Shi et al. used *Lactobacillus* strains to rebuild antibiotic–induced dysfunction in the gut microbiota and achieved promising results [26]. The administration of Lactobacillus paracasei DG could protect children with autism [27], HIV infected persons [28], and patients with irritable bowel syndrome [29,30] by increasing the ratio of *Blautia*: *Coprococcus*. Our present study also showed a similar trend: *L. plantarum* CCFM8610 could reduce the changes in intestinal flora structure caused by exposure to different foodborne forms of Cd. Considering that different foodborne forms of Cd could lead to distinct changes in the intestinal flora, it is an interesting finding that deserves further study for its relevant mitigation mechanisms.

Serum levels of metabolites are associated with Cd toxicity. Kato et al. treated rats with CdCl_2_ at a low dose for 90 days and found that amino malonic acid was a biomarker for the toxicity of Cd alone [31]. Jia et al. also found that quercetin could relieve Cd toxicity by regulating the metabolism of lipids and amino acids [32]. In this study, the effects of different foodborne forms of Cd treatment on serum metabolites were investigated. The results showed that the Cd used in the experiment could cause significant changes in the serum metabolites of mice, which mainly involves amino acids and fatty acids. It is interesting to find that different foodborne forms of Cd resulted in different changes in the serum metabolite content, which corresponds to the microflora results.

Many studies have shown that the gut microflora is closely correlated with serum metabolites, which can affect physical health. Liu et al. compared serum metabolomics and the diversity of intestinal flora of lean and obese individuals. A significant decrease in *Bacteroides thetaiotaomicron* was observed in the obese population, and correlation analysis showed that *Bacteroides thetaiotaomicron* had a strong association with serum glutamate [33]. In this study, the relationship between the gut flora and metabolites under the treatment of different foodborne forms of cadmium and CCFM8610 was investigated. The arginine biosynthesis, d–glutamine and d–glutamate metabolism, and fructose and mannose metabolism were involved in all Cd treatment groups, which indicated that the pathways might be important for the remission of Cd toxicity.

Probiotics could specifically adsorb cadmium [34], and protect the gut barrier by adjusting the structure of intestinal flora [16], resulting in the reduction in cadmium accumulation in the body. Meanwhile, the reduction in cadmium exposure in vivo and the changes in metabolic function of intestinal flora can then regulate the metabolic activities of the body and alleviate the oxidative stress caused by cadmium exposure [35]. A similar phenomenon was found when we explored the mechanism of CCFM8610 alleviating the toxicity of different foodborne cadmium. For example, the relative abundance of *Anaerostipes*, which has been reported to have a negative correlation with cadmium accumulation [36], decreased after the exposure to Cd–GSH. The administration of CCFM8610 limited this change, and the correlation analysis showed that *Anaerostipes* has a positive association with spermidine, which has been reported to migrate cadmium toxicity to wheat [37]. The changes in serum spermidine concentration in Cd–GSH treatment groups also confirmed this correlation (Table S1). These findings indicate that CCFM8610 could limit the changes in the gut microbiota, so as to affect the accumulation of cadmium in the body and alleviate the toxicity of cadmium by influencing serum metabolism. Additionally, our previous studies suggested that the toxic effects caused by Cd are mainly associated with free Cd^2+^ content dissociated in the gastrointestinal tract; however, the diverse changes in the gut microflora and serum metabolites in different groups may indicate that the toxic effects caused by the different foodborne forms of Cd may not only be limited to free Cd^2+^. The ligand of Cd may also play a key role in their toxicity mechanism, which deserves further investigation and verification tests.

## 4. Materials and Methods

### 4.1. Preparation of Diverse Foodborne Forms of Cadmium

CdCl_2_ was purchased from Sangon Biotech Co. Ltd., Shanghai, China. The preparation of Cd–citrate, Cd–GSH, and Cd–MT were performed as described in previous studies [14,38].

### 4.2. Preparation of L. plantarum CCFM8610

*L. plantarum* CCFM8610 was cultured, separated, and lyophilized in skim milk. Freeze–dried cells were resuspended at a concentration of 1 × 10^10^ CFU/mL for further use.

### 4.3. Animal Treatment

Forty SPF male ICR mice were purchased from JOINN Laboratories, Suzhou, China. The test mice were weighed and randomly divided into 10 groups (four mice per group). The mice were housed for 1 week without any treatment, and then treated with CdCl_2_, Cd–citrate, Cd–MT, and Cd–GSH solution separately by gavage. The dose of Cd was 50 mg Cd^2+^/kg bw, which had been found to cause no death in mice but has toxic effects on organs. One hour after Cd administration, the treatment groups were treated with CCFM8610, and the other groups were treated with skim milk as a control. All mice were housed in a barrier environment and the room temperature was maintained at 23 °C.

All mice were sacrificed 48 h after the oral administration. Blood samples were collected from each mouse. The renal tissues and hepatic tissues were collected and preserved in a −80 °C refrigerator for further analysis. The animal experiments were approved by the Experimental Animal Ethics Committee of Jiangnan University (JN.No20180615i0800723 [167]).

### 4.4. Determination of Cadmium Content

Four samples per group were weighed, and 5 mL of HNO_3_ was then added for cold digestion overnight. The mixture was diluted to 10 mL with deionized water after complete digestion using a microwave digestion system. The Cd content was determined using an atomic absorption spectrometer.

### 4.5. Analysis of Antioxidant Indexes in Livers and Kidneys

Analysis of antioxidant indexes in the liver and kidneys was mainly based on a previous study [39]. The assay kits (Nanjing Jiancheng Bioengineering Institute, Nanjing, China) were used to determine the concentrations of MDA, as well as the activity of superoxide dismutase (SOD) and catalase (CAT) in the liver and kidneys. Hepatic MT concentration was determined by the elisakit produced by Shanghai Enzyme–linked Biotechnology Co., Ltd., Shanghai, China. The total protein concentration was determined using an assay kit that was purchased from Beyotime, Shanghai, China.

### 4.6. Analysis of Fecal Microbial Diversity

Analysis of fecal microbial diversity based on 16S rRNA gene sequencing technology has been described in a previous study [40]. Briefly, the FastDNA Spin Kit for Feces (MP Biomedical, Solon, OH, USA) was used for DNA extraction. The V4 region of the 16S rRNA gene was amplified and purified. Sequencing was performed on a MiSeq sequencer platform at Jiangnan University.

### 4.7. Metabolomics Analysis

To perform metabolomic analysis, 100 μL of serum was pipetted into a 1.5 mL centrifuge tube, then 400 μL of methanol (precooled at −80 °C) was added to precipitate the protein. The samples were vortexed for 30 s and placed in a refrigerator at −20 °C for 1 h to increase the protein precipitation rate. The samples were then centrifuged at a high speed at 12,000 rpm for 15 min at 4 °C. Next, 400 μL of the supernatant was taken and evaporated to dryness at 4 °C. One hundred microliters of methanol:water (8:2) was added to reconstitute the samples, followed by vortexing and centrifugation as described above. The QC sample was prepared by transferring an equal volume from the samples to be tested into a new tube [41].

The metabolomics samples were tested using the Q Exactive Orbitrap Mass Spectrometer system. LC separation was performed using an Acquity HSS T3 column (Waters, 1.8 μm, 2.1 × 100 mm). The flow rate of the mobile phase was 0.3 mL/min with an injection volume of 2 μL over a 15 min runtime. Blank and QC samples were also analyzed. Chromatographic peak alignment and metabolite annotation were achieved using Compound Discover 3.0.

All samples were normalized by excluding the blank background and a coefficient of variation of >30% in the QC samples. All metabolic compound data were prepared according to SIMCA–P (V14.1) for orthogonal partial least squares discrimination analysis (OPLS–DA). The differential metabolites were screened using one–way ANOVA to determine *p*–values (*p* < 0.05) among the groups. MetaboAnalyst 5.0, was used to identify the metabolite–related pathways.

### 4.8. Statistical Analysis

Statistical analyses were performed using SPSS (V23). Briefly, the homogeneity of variance and normal distribution analysis of the data were first performed [42]. One–way analysis of variance followed by Tukey’s post hoc test was then selected to analyze the difference among groups, and a value of *p* < 0.05 was defined as a significant difference between comparisons.

## 5. Conclusions

In this study, Cd–GSH, Cd–citrate, CdCl_2_, and Cd–MT were used to establish an acute toxicity model in mice to explore the ability of CCFM8610 to relieve the toxic effects caused by different foodborne forms of Cd. By measuring the accumulation of Cd in the body and changes in the antioxidant system, it was found that probiotics have a promising effect on the reduction in the biological toxicity effect of different foodborne forms of Cd. At the same time, the protective effect of intestinal microflora and serum metabolites might be an important mechanism for probiotics to alleviate Cd toxicity.

## Data Availability

All data presented in this study are available in the main body of the manuscript.

## Supplementary Materials

The following are available online at https://www.mdpi.com/article/10.3390/ijms222011045/s1.
