# Supplementary material for: Protective Effects of Lactobacillus plantarum CCFM8610 against Acute Toxicity Caused by Different Food-Derived Forms of Cadmium in Mice
Source: Int J Mol Sci. 2021 Oct 13;22(20):11045. doi: 10.3390/ijms222011045 (PMC8537435; doi:10.3390/ijms222011045)
Supplement: Supplementary file 1 [file ijms-22-11045-s001.zip › ijms-1397520-supplementary.pdf]

Table S1. Relative differences in identified metabolite levels in mice.

	Relative ion intensity (10^5)										P values									Class
	Control		$CdCl_2$		Cd-Cit		Cd-GSH		Cd-MT		Control	$CdCl_2$		Cd-Cit		Cd-GSH		Cd-MT		
	C	8610	Cd	Cd8	Cit	Cit8	GSH	GSH8	MT	MT8	C-8610	Cd-C	Cd8-Cd	Cit-C	Cit8-Cit	GSH-C	GSH8-GSH	MT-C	MT8-MT	
A1	128	83	128	114	100	142	136	121	126	127	0.129	0.936	0.109	**0.027**	**0.031**	0.751	0.692	0.927	0.971	
A2	181	136	187	146	166	144	166	177	198	167	0.170	0.784	0.183	0.379	0.290	0.699	0.769	0.091	**0.025**	
A3	45	37	33	54	46	46	28	59	31	27	0.689	0.344	0.256	0.932	0.972	0.232	**0.029**	0.284	0.346	Amines
A4	108	62	97	78	67	60	64	91	70	100	0.086	0.666	0.439	**0.008**	0.644	0.212	0.396	**0.024**	0.054	
A5	137	122	182	191	217	189	140	152	151	163	0.665	**0.042**	0.883	**0.039**	0.546	0.921	0.745	0.510	0.637	
A6	418	267	259	234	301	240	245	229	195	172	0.059	0.050	0.421	0.103	0.130	**0.041**	0.708	**0.017**	0.646	
A7	896	792	1,027	1,042	1,521	1,781	1,066	1,713	1,251	1,753	0.571	0.455	0.880	**0.027**	0.317	0.704	0.217	0.155	0.144	amino acid
A8	363	404	389	510	593	618	570	572	439	530	0.394	0.544	0.078	**0.001**	0.719	0.106	0.985	0.354	0.349	
A9	1,477	1,481	1,100	2,286	2,107	2,121	2,094	1,595	1,202	1,124	0.990	0.080	0.086	**0.016**	0.939	0.161	0.287	0.193	0.698	
A10	386	410	338	601	633	615	586	496	372	404	0.756	0.354	0.052	**0.008**	0.796	**0.022**	0.275	0.843	0.698	amino acid derivative
A11	163	195	168	231	171	273	260	280	265	299	0.258	0.702	0.257	0.642	**0.025**	0.194	0.816	0.067	0.547	
A12	21,621	24,485	18,075	21,671	19,906	16,794	13,984	13,720	16,737	16,399	0.137	0.158	0.375	0.641	0.545	0.151	0.949	**0.047**	0.833	
A13	96	78	82	80	68	54	52	64	66	60	0.287	0.236	0.775	**0.044**	0.090	**0.010**	0.158	0.161	0.710	
A14	4,937	4,200	3,318	3,265	3,446	2,770	2,925	2,553	3,150	2,607	0.316	**0.001**	0.816	0.061	0.377	0.124	0.695	0.059	0.471	
A15	1,677	1,439	978	795	1,198	872	1,080	728	950	679	0.415	**0.025**	0.159	0.175	0.365	0.315	0.520	0.077	0.416	
A16	19,094	17,233	9983	12,094	11,614	9,882	9,533	10,177	12,278	8,594	0.437	**0.003**	0.141	**0.004**	0.398	**0.025**	0.795	0.106	0.313	
A17	2,024	1,575	1,350	1,025	1,126	935	857	942	1,607	777	0.069	**0.005**	**0.018**	**0.002**	0.142	**0.019**	0.758	0.364	0.118	fatty acid
A18	29,404	25,705	20,120	25,506	27,281	25,050	21,652	18,123	21,604	17,035	0.354	**0.003**	0.069	0.312	0.698	0.177	0.466	**0.042**	0.161	
A19	6,624	7,093	4,935	5,741	5,158	4,001	3,731	4,213	5,284	4,064	0.478	**0.047**	0.329	0.072	0.115	**0.006**	0.354	0.218	0.238	
A20	75,714	72,186	58,943	70,000	64,905	56,763	51,155	54,146	58,631	51,387	0.528	**0.020**	0.120	0.173	0.454	0.069	0.748	**0.047**	0.306	
A21	1,435	1,154	1,191	949	1,046	739	733	944	964	878	0.314	0.235	0.348	0.060	**0.030**	**0.009**	0.116	0.145	0.742	

A22	517	477	487	477	423	422	445	461	450	424	0.248	0.371	0.754	**0.020**	0.958	0.268	0.793	0.111	0.497	
A23	132	170	88	141	158	153	130	128	119	113	0.331	0.053	**0.040**	0.214	0.803	0.872	0.898	0.264	0.560	Carboxylic Acids
A24	70	32	26	31	25	15	10	13	78	17	0.176	0.133	0.671	0.129	**0.035**	0.068	0.589	0.916	0.414	
A25	36	41	63	64	76	75	55	76	58	70	0.252	**0.000**	0.837	**0.024**	0.926	0.157	0.109	0.230	0.484	phospho sugar
A26	24	25	39	16	17	12	13	19	28	21	0.929	0.301	0.166	0.274	**0.026**	0.128	0.320	0.754	0.557	
A27	2,105	1,537	2,350	1,580	1,358	1,459	1,533	1,760	2,078	1,774	0.152	0.414	**0.022**	**0.050**	0.808	0.356	0.697	0.966	0.623	Vitamins
A28	158	96	130	158	132	139	118	119	91	122	0.108	0.277	0.329	0.212	0.789	0.219	0.955	**0.020**	0.079	
A29	8,473	6,966	9,598	8,266	9,511	9,596	8,906	9,233	8,767	9,443	0.521	0.486	**0.022**	0.560	0.941	0.822	0.827	0.888	0.728	uronic acid
A30	57	53	52	53	54	43	45	57	46	61	0.651	0.404	0.913	0.606	**0.017**	0.082	0.125	0.116	**0.014**	
A31	72	113	161	184	139	116	184	124	69	104	0.089	0.137	0.658	**0.027**	0.335	0.299	0.542	0.896	0.136	indoles
A32	100	118	42	205	192	214	147	143	159	89	0.775	0.315	**0.036**	0.170	0.662	0.413	0.958	0.410	0.228	alcohol
A33	82	85	58	48	53	24	37	58	47	56	0.810	0.247	0.621	0.204	0.191	**0.030**	0.134	**0.022**	0.599	Cresols
A34	4,319	5,473	3,365	5,642	5,866	5,891	4,329	5,507	4,205	4,744	0.210	**0.028**	0.057	0.060	0.980	0.988	0.316	0.776	0.193	Eicosanoids
A35	817	511	730	698	553	663	384	590	703	602	0.201	0.408	0.803	**0.025**	0.224	0.138	0.424	0.445	0.489	purines
A36	1,899	1,679	1,074	1,521	949	1,273	828	1,286	1,200	940	0.475	0.053	0.170	**0.024**	0.282	**0.015**	0.109	0.064	0.406	D-Galactose
A37	565	498	615	516	526	452	478	473	493	630	0.100	0.150	**0.030**	0.314	0.129	0.392	0.961	0.197	0.105	Sulfuric Acids

The differently shaded cells of the table meant to represent the difference of relative ion intensity. A1, 3-Methylhistamine; A2, Acetylcholine; A3, Histamine; A4, Spermidine; A5, Sphingosine; A6, Citrulline; A7, L-Arginine; A8, L-Glutamic acid; A9, L-Methionine; A10, Methionine sulfoxide; A11, Taurine; A12, 16-Hydroxy hexadecanoic acid; A13, 2-Furoic acid; A14, 8,11,14-Eicosatrienoic acid; A15, Adrenic acid; A16, Docosahexaenoic acid; A17, Eicosapentaenoic acid; A18, Elaidic acid; A19, Ethyl dodecanoate; A20, Ethyl tetradecanoate; A21, Oxoglutaric acid; A22, Pelargonic acid; A23, Ginkgoic acid; A24, Urocanic acid; A25, Beta-D-Fructose 6-phosphate; A26, Ribose 1-phosphate; A27, L-Acetylcarnitine; A28, Pantothenic acid; A29, L-Norleucine; A30, Beta-D-Glucopyranuronic acid; A31, Indoxyl sulfate; A32, Corticosterone; A33, 2,6-Di-tert-butyl-4-hydroxymethylphenol; A34, 11,12-EpETrE; A35, Uric acid; A36, D-Galactose; A37, Sulfate.

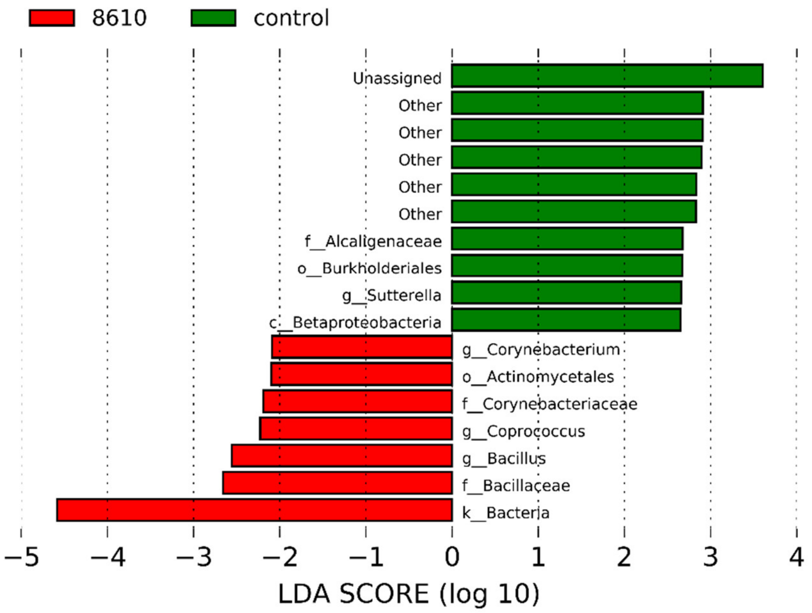

Figure S1. LDA effect size (LEfSe) analysis results of fecal microbiota in CCFM8610 treated groups.

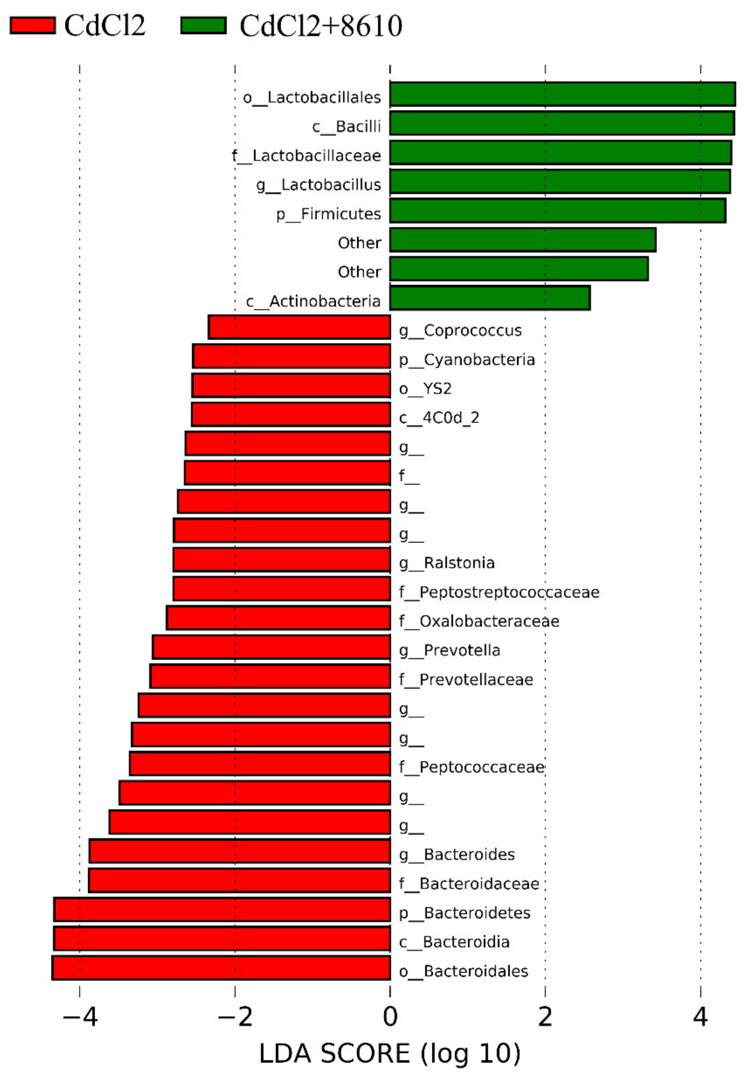

Figure S2. LDA effect size (LEfSe) analysis results of fecal microbiota in $CdCl_2$ treated groups.

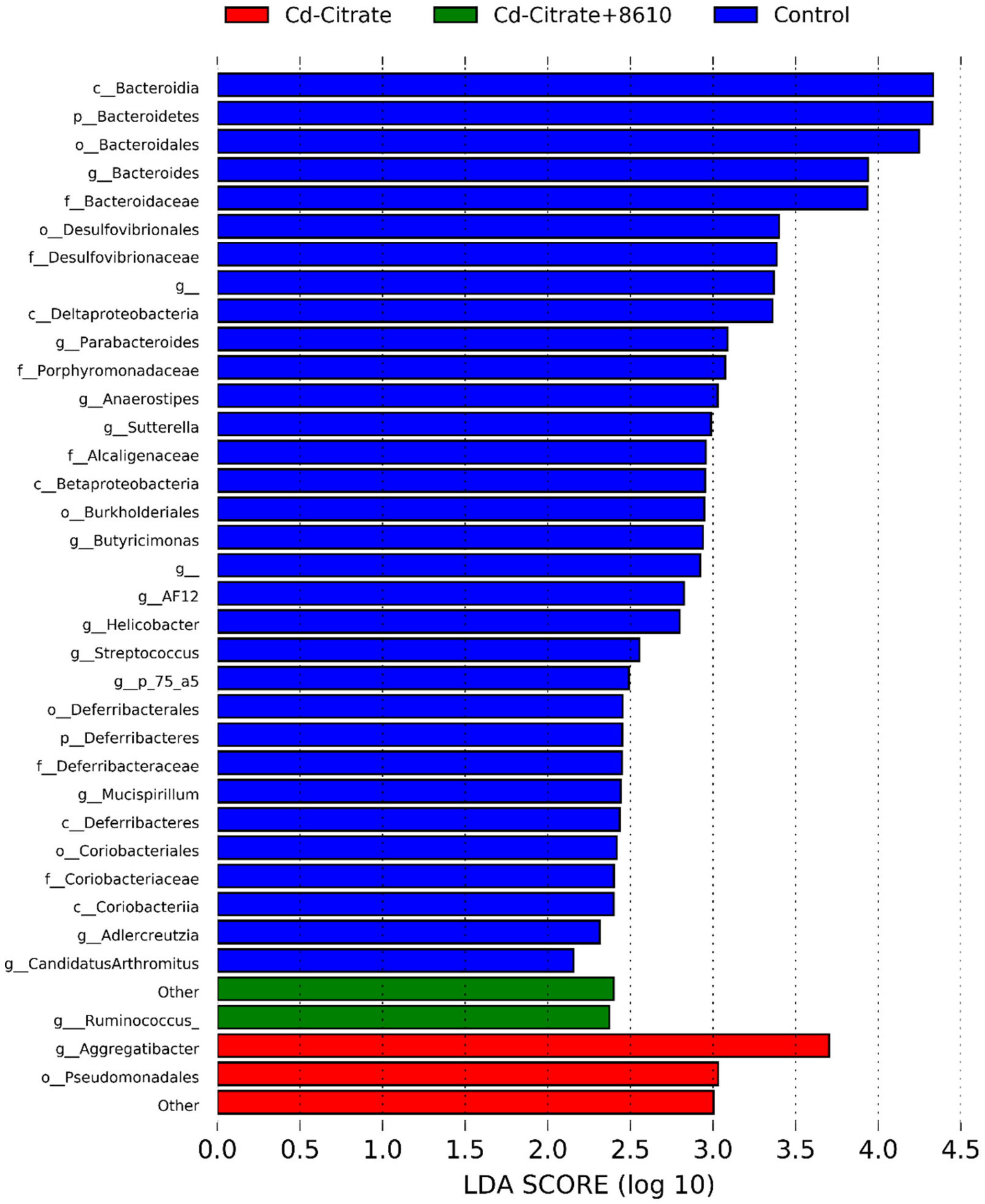

Figure S3. LDA effect size (LEfSe) analysis results of fecal microbiota in Cd-Citrate treated groups.

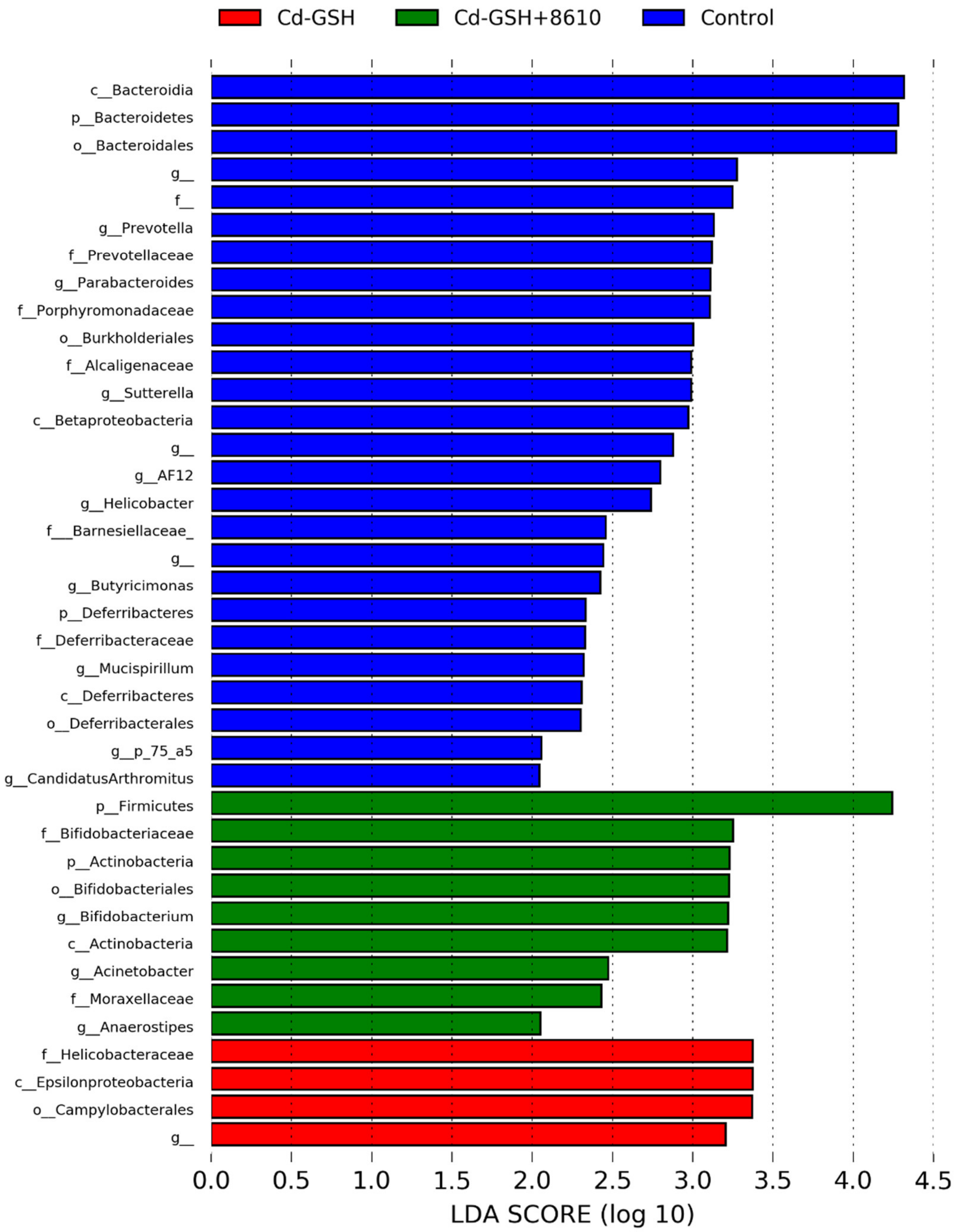

Figure S4. LDA effect size (LEfSe) analysis results of fecal microbiota in Cd-GSH treated groups.

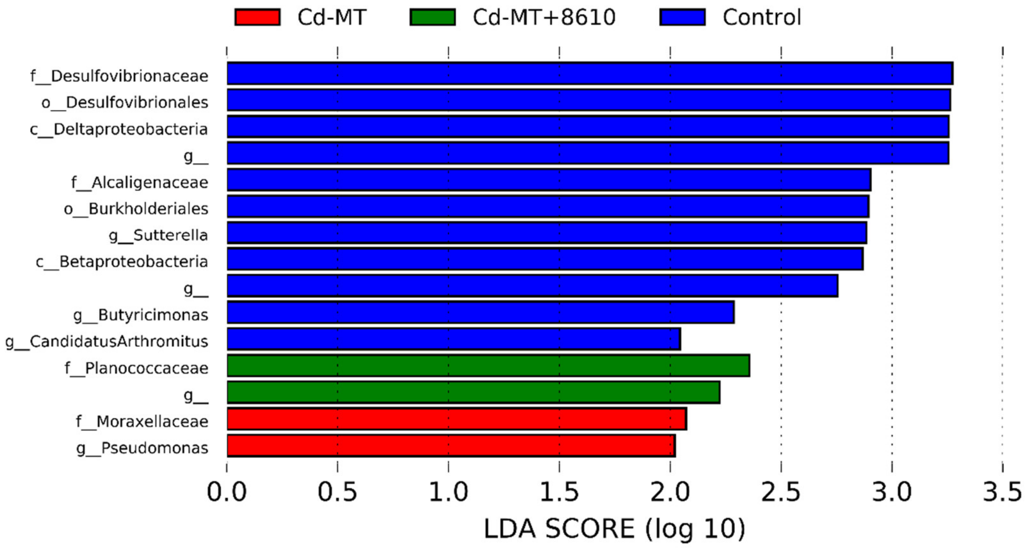

Figure S5. LDA effect size (LEfSe) analysis results of fecal microbiota in Cd-MT treated groups.
